# The Battle of RNA Synthesis: Virus versus Host

**DOI:** 10.3390/v9100309

**Published:** 2017-10-21

**Authors:** Alex Harwig, Robert Landick, Ben Berkhout

**Affiliations:** 1 Department of Biochemistry, University of Wisconsin-Madison, Madison, WI 53706, USA; harwig@wisc.edu; 2 Laboratory of Experimental Virology, Department of Medical Microbiology, Center for Infection and Immunity Amsterdam (CINIMA), Academic Medical Center, University of Amsterdam, Meibergdreef 15, 1105 AZ Amsterdam, The Netherlands

**Keywords:** transcription, nascent RNA, HIV-1, influenza, Epstein-Barr virus, RNA polymerase II, RNA-dependent RNA polymerase, respiratory syncytial virus

## Abstract

Transcription control is the foundation of gene regulation. Whereas a cell is fully equipped for this task, viruses often depend on the host to supply tools for their transcription program. Over the course of evolution and adaptation, viruses have found diverse ways to optimally exploit cellular host processes such as transcription to their own benefit. Just as cells are increasingly understood to employ nascent RNAs in transcription regulation, recent discoveries are revealing how viruses use nascent RNAs to benefit their own gene expression. In this review, we first outline the two different transcription programs used by viruses, i.e., transcription (DNA-dependent) and RNA-dependent RNA synthesis. Subsequently, we use the distinct stages (initiation, elongation, termination) to describe the latest insights into nascent RNA-mediated regulation in the context of each relevant stage.

## 1. Coevolution of Viral and Host mRNA Synthesis Pathways

All living organisms are fully equipped to synthesize messenger RNA (mRNA) using their endogenously encoded multi-subunit RNA polymerases (RNAPs). However, many viruses form an exception to this dogma and employ a parasitic lifestyle that exploit parts of the host transcription pathways. This co-option allows viruses to dramatically downsize their genomes to the minimum number of genes essential for successful infection. As a result, viruses and their hosts have been involved in an eternal battle of adaptation and counter-adaptation for RNA synthesis since far before the emergence of humans [1,2,3]. During this time, viruses coevolved many pathways to transcribe their own genetic material, meanwhile avoiding counter-adaptations of the host [4]. Weapons in the arsenal of the virus include virally-encoded proteins and micro RNAs (miRNAs), which alter transcription pathways in both host and virus via many different mechanisms. These mechanisms have been reviewed extensively elsewhere (for example [5,6,7,8]). However, one major, yet underappreciated, component of viral gene regulation occurs via nascent RNAs.

RNAs have many attractive properties that can be used by viruses to manipulate gene regulation pathways. First, RNAs, like DNAs, can specifically pair with complementary viral or host sequences using minimal investment of genetic material, thus allowing further minimization of the viral genome. Encoding a DNA-binding protein, on the other hand, consumes significantly more genetic information. A typical DNA-binding domain recognizes ~10 base pairs (bp) or more of DNA and comprises at least ~60 amino acids (aa), thereby requiring 180 bp of encoding DNA [9]. In contrast, RNA requires only the number of complementary nucleotides as the DNA/RNA region it recognizes. This can be beneficial for the virus where space constraints are dictated by the size of the viral capsid. Second, RNAs are less immunogenic compared to proteins. Whereas hosts developed many pathways to respond to foreign invading proteins, RNAs are dealt with by relatively easy to avoid RNAi machinery or certain Toll-like and retinoic acid-inducible gene I (RIG-I)-like receptors [10]. Furthermore, because RNAs can be smaller (see point one) it is easier to avoid recognition by the host’s immune system. Third, RNA can store information in its sequence, its secondary and tertiary structure, its ability to form ligand-binding platforms (e.g., riboswitches or ribozymes), and via chemical modification of bases and sugars. This makes RNA a flexible molecule, adaptive to multiple situations. Fourth, RNAs, like proteins, are modular and can use domains or different surfaces within one domain to interact with other molecules [11]. The modular nature of RNAs and the versatility of each module (see point three) dramatically expands the repertoire of regulatory RNAs.

For decades, the principal focus of gene regulation studies has been on protein regulators, but recent discoveries in virology highlight the ever-increasing appreciation that RNAs, including nascent RNAs, also play a major part in gene regulation by viruses. In this review, we will describe several recent discoveries of intriguing viral pathways that manipulate gene expression of the host for their own benefit via nascent RNA. To place these discoveries in context, we will first describe the current understanding of the distinct stages (initiation, elongation, termination) in the general mechanism of eukaryotic transcription, as used by the host and virus, and present examples of nascent RNA-mediated regulation in the context of each relevant stage.

## 2. The Viral RNA Synthesis Machinery

**Figure 1:**
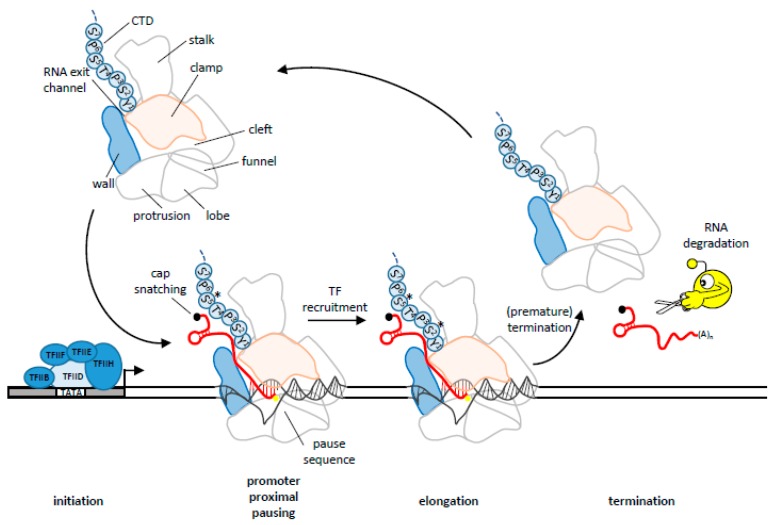
Transcription cycle. A cartoon depiction of RNA polymerase II (RNAPII) is shown in the upper left, with several domains and modules labelled. The clamp and wall (flap in prokaryotes) modules that make up the RNA-exit channel are depicted in pink and blue, respectively. The first repeat of the RNAPII C-terminal domain (CTD) is shown as a string of amino acids with their respective numbers. Next, the stages of the transcription cycle are shown, with the viral interfering mechanisms. First, general transcription factors assemble at the promoter and direct RNAPII towards the transcription start site. Transcription factor (TF) IIH phosphorylates the RNAPII at Ser5 (black asterisk). TFIIH opens the DNA template, forming the transcription bubble, permitting RNAPII to begin transcribing RNA in its active center (yellow dot). Upon transcribing the first 20–60 nucleotides, most RNAP will pause. Transcription factor recruitment regulates these transcription kinetics and phosphorylate RNAPII at Ser2. When the correct signal is encountered, RNAPII will terminate transcription and release the RNA. Some viruses encode endonucleases (yellow sphere with scissor) that can cleave RNAs, causing RNA degradation.

Cellular RNA is synthesized from a DNA template in a process called transcription by RNAP-containing transcription complexes. Eukaryotes employ three different classes of RNAP: RNAPI, II, III, while plants also encode for RNAPIV and V [12]. Of these, primarily RNAPII is used to synthesize cellular mRNA and viral mRNAs. RNAPII is a 550 kDa multi-subunit enzyme complex. These subunits are arranged in a claw-like manner (Figure 1), with a central cleft through which the template DNA is threaded. Nucleoside triphosphates (NTPs) enter the polymerase via the funnel and the newly transcribed RNA exits RNAPII through an RNA-exit channel formed by the mobile clamp and wall elements. Unique to RNAPII is an unstructured carboxy-terminal domain (CTD) that extends from the largest subunit and is composed of 52 heptapeptide repeats (consensus: Tyr^1^-Ser^2^-Pro^3^-Thr^4^-Ser^5^-Pro^6^-Ser^7^) [13]. The serine, threonine, and tyrosine residues can be reversibly phosphorylated [14]. Changes in the phosphorylation patterns of the CTD during transcription orchestrate the association of different sets of transcription-regulating factors with RNAP.

In the early 1960s, researchers observed that a subset of viruses were resistant to actinomycin D, a drug that inhibits cellular DNA-directed RNA synthesis as performed by RNAPII [15]. This led to the discovery of RNA viruses. Like their DNA-containing counterparts, these RNA viruses are grouped into double stranded (ds) and single stranded (ss) virus classes. The ssRNA viruses are further subdivided into negative or positive sense single-stranded (−ss or +ss, respectively), depending on viral genomic RNA (vRNA) polarity. All RNA viruses encode an RNA-dependent RNA polymerase (RdRp) to copy the vRNA and synthesize the viral mRNA. RdRp synthesizes RNA complementary to the starting RNA template and is an essential protein of all RNA viruses that have no intermediate DNA-stage [16,17]. This process is often called “transcription”, but differs from the conventional definition of transcription by using RNA rather than DNA as a template for mRNA synthesis. To avoid confusion, we will refer to RNA-dependent mRNA (Rd-mRNA) synthesis in this review. Both transcription and Rd-mRNA synthesis occur in three stages: initiation, elongation and termination (Figure 1). During initiation, the polymerase machinery is recruited to the viral promoter. Initiation of Rd-mRNA synthesis begins at or near the 3′ end of the RNA template in a primer-independent (de novo), or a primer-dependent mechanism. Subsequently, 5′ to 3′ elongation starts but is frequently interrupted by pauses [18]. Finally, the mRNA is terminated and released from the elongation complex. During the elongation phase, a complementary mRNA product is generated by 5′ to 3′ processive nucleotidyl transfer [19,20]. The three stages of mRNA synthesis by both RNAP and RdRp, and their associated regulatory events, are described in detail below.

## 3. Transcription Initiation

Initiation is promoted by factors that recruit RNAP to the promoter, melt the DNA duplex and load the template DNA strand into the active site of RNAP. For RNAPII, these steps require the combined action of basal transcription factors (TF) (Figure 1) [21,22]. The minimal pre-initiation complex (PIC) includes RNAPII and six general transcription factors: TFIIA, TFIIB, TFIID, TFIIE, TFIIF, and TFIIH. First, TFIID containing the TATA-binding protein (TBP) is recruited to TATA box containing promoter sequences (Figure 1) [23,24,25]. The binding of TFIID to the TATA box in the promoter region of the gene initiates the recruitment of TFIIB, TFIIE, TFIIF and TFIIH, which position the double-stranded (ds) DNA above the cleft within RNAPII [26,27,28]. Local unwinding of the dsDNA by the Ssl2 helicase subunit of TFIIH delivers the template strand into the RNAPII active center and creates a DNA bubble downstream of the TATA box [29]. Efficient DNA opening requires TFIIE and TFIIH, but these factors are not required for low levels of transcription [30]. The TFIIK kinase module of TFIIH will subsequently phosphorylate RNAPII on the Ser5 of its CTD repeats, facilitating promoter clearance and starting the elongation stage [31].

## 4. Initiation of RNA-Dependent RNA Synthesis

Whereas DNA viruses only need to generate mRNA, RNA viruses without a DNA stage have to synthesize both vRNA and mRNA. The vRNA is generated through a replication intermediate, named antigenome which serves as a template for vRNA synthesis. ssRNA viruses evolved sophisticated approaches to generate both RNA species from one template. Using respiratory syncytial virus (RSV) as a model, it was illustrated that RdRp has two RNA synthesis activities which determine nascent RNA fate. RSV is a −ssRNA virus and the major cause of respiratory tract disease in infants and young children, causing between 66,000 and 199,000 deaths per year worldwide [32]. To differentiate between Rd-mRNA synthesis and vRNA replication, RdRp initiates at one of two different start sites within the promoter sequences at the 3′ end of the RSV RNA genome. Synthesis of the antigenome starts at the expected +1 site. However, the more abundant RNA species in infected cells was found to initiate at the +3 site [33]. RdRp’s that initiate at the +3 site are unable to enter a stable elongation mode and release the RNA after approximately 20–25 nucleotides. Many de novo transcription initiation events by RdRp are known to generate abortive transcripts during the initiation phase of RNA synthesis [34,35,36]. However, the function of this small RNA is yet unknown. It has been proposed that synthesis of this small abortive RNA allows the polymerase to break contacts with the promoter [37]. After releasing the small RNA, the RSV polymerase can now scan the template and locate the conserved gene start signal (~10–13 nt) of the first gene, however the mechanism behind this transition is unknown [38]. Here the polymerase will reinitiate RNA synthesis, cap the 5′ end of the mRNA and commit the RdRp to Rd-mRNA synthesis. The choice between the +1 and +3 initiation site by RSV RdRp seems to be determined by the loading of a specific nucleotide and subsequent creation of a dinucleotide primer complementary to the specific initiation site [37]. Remarkably, even when the priming site is mutated or absent, the enzyme is able to selectively synthesize the original dinucleotide priming site at the 3′ end of the viral RNA, explaining the strict conservation of the 5′- and 3′-end dinucleotides of both +ss and −ssRNA viruses [39]. It has been proposed that a domain, unique in de novo initiating RdRp’s, named “priming loop” (T794 to A799) regulates the accessibility of the active site of RdRp [40]. An essential role during initiation is played by the residue H798 of the priming loop, by providing a platform to which the base of the priming ATP could establish a stacking interaction [39]. These conservational mechanisms, mediated by the polymerase alone have been suggested to be used by several ssRNA viruses [41,42].

## 5. How to Obtain a Cap?

**Figure 2:**
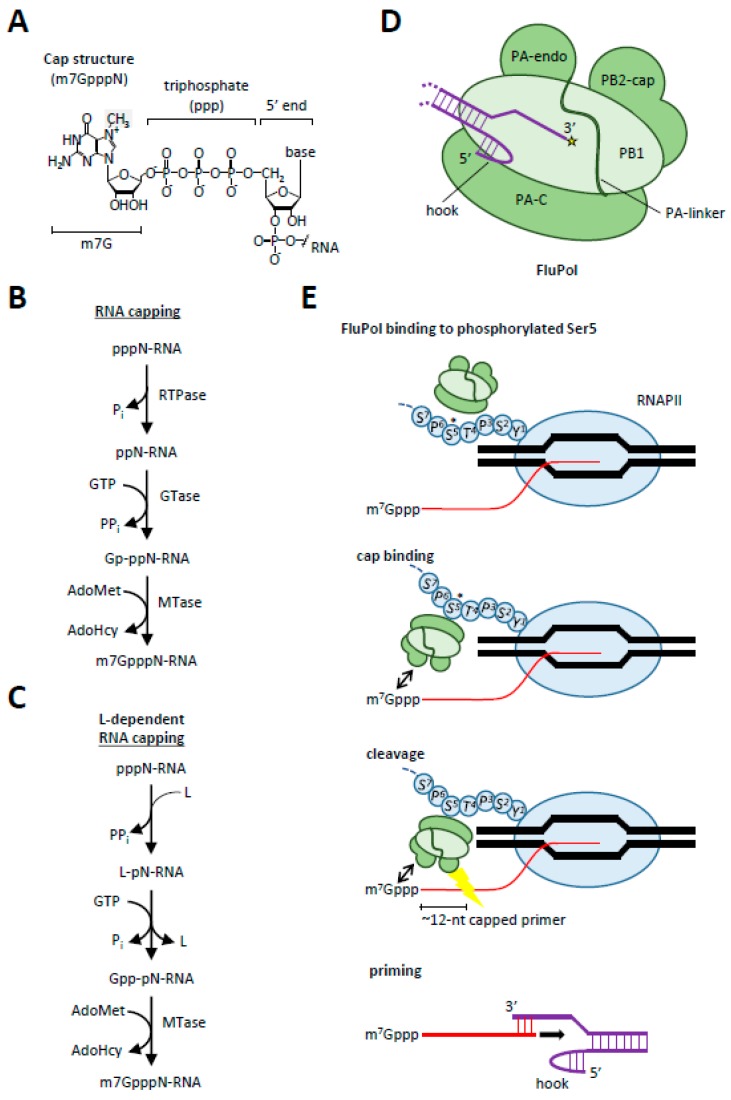
Capping mechanisms in RNA viruses. (**A**) The eukaryotic mRNA cap consists of a 7-methylguanosine linked to the initiator nucleoside of mRNA through the 5′-5′ triphosphate bridge. The methyl group at the N7 position of the guanosine is shaded gray; (**B**) the conventional RNA capping pathway; (**C**) the L-dependent capping pathway utilized by negative-sense ssRNA viruses; (**D**) the FluPol complex with the polymerase acidic endonuclease (PA-endo), PA C-terminal (PA-C), polymerase basic 2 cap-binding (PB2-cap) and PB1 domains indicated. The influenza viral RNA (vRNA; purple) is circularized with the extreme 5′ end docked in a pocket formed by PA-C and PB1 and the 3′ end loaded in the FluPol active center (yellow star); (**E**) the first step in cap-snatching is the recognition and binding of FluPol to the phosphorylated Ser5 of RNAPII through its PA-C domain. This will allow the PB2-cap domain to bind the cap and to direct the nascent RNA towards PA-endo. After cleavage the nascent RNA will be loaded into the active domain of FluPol where it will be used to prime the 3′ end of the vRNA, allowing elongation of the RNA primer from 5′ to 3′ direction (indicated with black arrow).

5′-terminal capping of eukaryotic mRNAs is probably the first co-transcriptional RNA processing event. Formation of the 5′-terminal cap structure (m7Gppp[5′]N-; where N is the first nt of the nascent RNA; Figure 2A) in eukaryotic mRNAs, in which 7-methylguanosine (m7G) is linked to the initiator nucleoside of mRNA through the 5′-5′ triphosphate bridge, is mediated by a series of enzymatic steps and is required for the efficient translation and stability of mRNAs. Eukaryotes and some DNA viruses (e.g., vaccinia virus) and dsRNA viruses (e.g., reovirus) obtain this cap by processing the 5′-triphosphate end (pppN-) of nascent RNA transcripts into a diphosphate (ppN-) using an RNA 5′-triphosphatase (RTPase) (Figure 2B). Subsequently, the guanosine monophosphate (GMP) moiety of a guanosine triphosphate (GTP) is transferred to the 5′ diphosphate by a GTP:RNA guanylyltransferase (GTase) to generate the cap core structure (Gp-ppN-). This core structure is then methylated at the guanine-7N position by *S*-adenosyl-l-methionine (AdoMet)-dependent cap (guanine-N7) methyltransferase (MTase) to produce the final cap structure (m7GpppN-). Higher eukaryotes and some viruses include an additional methylation step in which the 2′ OH position of the penultimate ribose is methylated by cap ribose-2′-*O*-MTase (m7GpppNm-).

However, ssRNA viruses (e.g., influenza, Ebola, measles) do not have a DNA template and thus cannot rely on the cellular capping enzymes. For this reason, RNA viruses have evolved capping mechanisms that are different from capping during cellular transcription. Most +ssRNA viruses (e.g., flaviviruses and nidoviruses) encode capping enzymes that selectively cap the newly synthesized viral mRNAs [43,44,45]. For instance, the flavivirus-encoded nonstructural protein 3 (NS3) contains a triphosphatase that releases the terminal phosphate from the 5′-triphosphate end of nascent + stranded RNA, forming a diphosphorylated ppN-RNA [46]. Subsequently, a GMP moiety from GTP is transferred to the 5′ end of the ppN-RNA through the GTase activity of the viral NS5 MTase. The GpppA-capped RNA is further methylated by the NS5 MTase to form the final m(7)GpppA- and m(7)GpppNm-RNA products [47,48]. However, both the process of discrimination between −RNA and +RNA 5′-ends for capping and the precise timing of the capping process remain largely undefined [49].

On the other hand, unsegmented −ssRNA viruses (including rabies, measles, and Ebola) carry an RdRp termed “L” and a phosphoprotein “P”, which share extensive amino acid sequence similarity among all these viruses [50]. P is an essential co-factor that targets the L polymerase to the vRNA [51,52,53]. Here, the L polymerase covalently links with the 5′-monophosphorylated viral mRNA-start sequence to form an intermediate complex (Figure 2C) [54]. Subsequently, the RNA:GDP polyribonucleotidyltransferase activity of the L polymerase transfers the GDP moiety of GTP to the monophosphorylated viral mRNA forming the viral mRNA cap. Thus, in contrast to GTases which transfer a monophosphate to the 5′ diphosphate end of RNA, the L polymerase-mediated capping reaction transfers a diphosphate to the 5′ monophosphate end of RNA.

Another solution to the capping problem of the negative-sense RNA viruses comes from the influenza virus in the form of “cap-snatching” from nascent host pre-mRNAs [55]. To synthesize viral mRNA and vRNA from each of the eight single-stranded viral RNA segments, influenza uses a virally encoded heterotrimeric RdRp called FluPol (Figure 2D). FluPol consists of three virally encoded subunits: polymerase basic 1 (PB1), PB2, and polymerase acidic (PA) arranged in a globular structure [56,57,58]. Each genome segment is circularized by the base-pairing of the near complementary 5′ and 3′ ends, and individually packaged into viral ribonucleoprotein particles (vRNPs) together with one copy of FluPol. FluPol is bound to the duplex at the point of circularization, tightly docked into the 5′ end of the vRNA by a pocket formed between the PA and PB1 subunits, creating an intramolecular stem-loop (‘hook’; Figure 2A) [56,57]. The 3′ segment of the negative-sense vRNA enters the active-site cavity of FluPol, where it serves as a template (Figure 2E) [59,60].

After entry into the cell, the vRNPs are released and transported into the nucleus. After arrival in the nucleus, the C-terminal domain of the FluPol PA subunit will specifically target and bind the phosphorylated Ser5 of actively transcribing RNAPII CTD [61] (Figure 2E). Subsequently, the PB2 cap-binding domain associates with the 5′ end of the cellular mRNA [60]. The PA endonuclease domain next cleaves the mRNA. This creates a capped 11–12 nt small RNA primer that is loaded into the active site of FluPol [62,63,64] (Figure 2E). Processive Rd-mRNA synthesis is initiated by the addition of an NTP to the 3′ end of the capped primer, complementary to the second or third residue in the vRNA template. During elongation, the 5′-capped viral mRNA is separated from the vRNA template by threading into their respective exit channels. After elongation, the viral mRNA is polyadenylated at an oligo-U stretch near the vRNA 5′ end [65]. The capped viral mRNA can now be used to translate all the necessary viral proteins, whereas the vRNA can be packaged, re-used as template, or degraded. Small host non-coding RNAs (snRNAs), especially U1 and U2 snRNA, are the preferred targets for cap-snatching, rather than mRNAs or pre-mRNAs [62,63,64]. Why the virus prefers to use these snRNAs as targets has yet to be experimentally established, but it has been proposed that the selective de-capping of U1 and U2 RNAs in combination with the binding of the viral NS1 protein to U6 snRNA may serve to inhibit host pre-mRNA splicing [66].

## 6. Transcription Elongation

Elongation is the repeated addition of a nucleoside monophosphate (NMP) to the 3′ end of the growing RNA chain. Progressive RNA synthesis during the elongation stage can be affected by several hurdles, including transcriptional pause sites, that RNAPII must overcome [67]. These transcriptional pauses can be used to create a time window for co-transcriptional events such as splicing, nucleotide modification, or RNA processing. Pauses also yield opportunities to regulate transcription in response to intra- and extra-cellular cues [68]. At most eukaryotic promoters, major transcriptional pauses typically occur within 20–60 nt after transcription initiation [69]. These promoter-proximal pauses are induced by a myriad of factors, including negative elongation factor (NELF) and nascent RNA structures. These pauses give opportunities for the organism to regulate transcription at an early time point.

## 7. Promoter-Proximal Paused RNA Transcripts as a Scaffold for Transcription Regulators

A promoter-proximal pause will often allow the nascent RNA to fold a structure that can serve as a protein-recruiting scaffold [70]. Additional transcription factors (TFs) can be recruited during this step. Three distinct modes of action can be envisaged whereby TF-interacting RNAs might affect transcription. They could act as (i) scaffolds to recruit one or more TFs [71,72,73]; (ii) competitors, regulating the binding or release of a TF [74]; or (iii) transacting guides for interacting TFs by base-pairing with DNA or RNA in the vicinity of their target sites [75].

**Figure 3:**
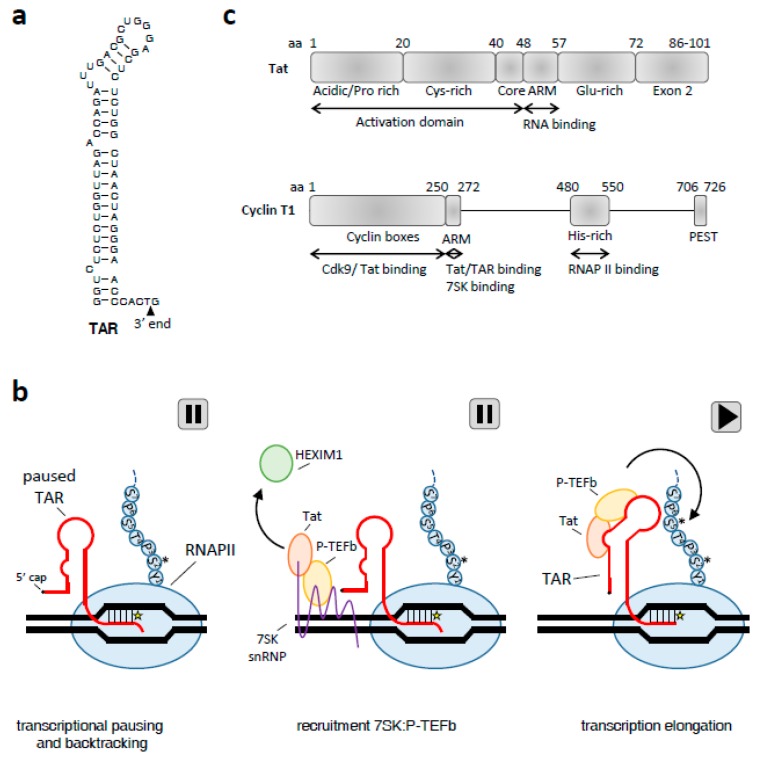
Factors involved in HIV-1 transcription and initiation. (**a**) Structure of the TAR hairpin; (**b**) model of RNAPII pausing (indicated with “II” button for pause) and reactivation (black triangle) upon TAR transcription. Phosphorylation of the CTD domain of RNAPII is indicated (black asterisk). The catalytic center of RNAPII is positioned at the yellow star. 7SK snRNA is depicted in purple. See the main text for more details; (**c**) domain organization of the viral Tat protein and positive transcription elongation factor b (P-TEFb) component Cyclin T1. Amino acid (aa) positions are indicated and the domain functions are described.

The best-studied case of a TF-recruiting RNA scaffold formed by promoter-proximal pausing is the human immunodeficiency virus type 1 (HIV-1) *trans*-acting region (TAR; Figure 3A) [76]. As a retrovirus, HIV-1 contains an RNA genome that is reverse transcribed into DNA upon entry of the host cell. This DNA integrates into the host genome as a provirus where it can be actively transcribed or enter a latent state. As in all retroviruses, the 5′ located long terminal repeat will act as the viral promoter. The viral promoter can be subdivided into the enhancer and core regions. The enhancer region contains binding sites for NF-κB [77,78], nuclear factor of activated T-cells (NFAT) [79], activator protein 1 (AP1) [80,81] and variable other TFs dependent on the HIV-1 subtype [82]. This enhancer upregulates HIV-1 transcription, but removal of its binding sites is not deleterious in certain cell lines [83]. In contrast, the core promoter is essential for transcription and contains three binding sites for SP1 [84] and a TATA box (CATATAA) element [81]. RNAPII, phosphorylated at Ser5 by TFIIH, will initiate transcription from this core region [85,86] (Figure 3B). However, most RNAPII complexes will pause after transcribing 62-nt, yielding short transcripts [87,88]. This is caused by the combined action of NELFs/5,6-dichloro-1-β-d-ribofuranosylbenzimidazole (DRB) sensitivity inducing factor (DSIF) [89,90], the local RNA structure [88,91], microprocessor [70] and sequences within template DNA that promote transcriptional pausing [92]. The position of the promoter-proximal paused RNAPII on the 3′ end of the short transcript allows the nascent TAR transcript to fold into an initial, semi-stable RNA structure [88] (Figure 3B; paused TAR). This nascent hairpin allows the initial RNAPII complex to backtrack [91], such that the 3′ end of the nascent transcript is displaced from the RNAPII catalytic center [93] (Figure 3B). As a result, RNAPII is paused until it is reactivated, which may include refolding into the most stable TAR configuration.

The folding of the TAR hairpin is key to the regulation of HIV-1 transcription [94] as it is used as a scaffold to recruit essential transcription factors, including the 86–101 amino acid (aa) viral *trans*-activator protein (Tat) [83] (Figure 3C). The interaction between Tat and TAR is necessary to recruit positive transcription elongation factor b (P-TEFb) from the 7SK small nuclear ribonucleoprotein (snRNP) [95]. P-TEFb consists of CyclinT1 (CycT1; Figure 3C) tightly associated with CDK9 kinase and is sequestered in an inactive form by the 7SK snRNP. Tat recruits P-TEFb by outcompeting the 7SK snRNP in several ways. First, the TAR-binding Arginine-rich motif (ARM) region of Tat (Figure 3C; aa 48–57) shows high similarity to the 7SK-binding of 7SK snRNP component hexamethylene bisacetamide-induced protein 1 (HEXIM1) [96]. Second, the HEXIM1-bound 7SK stem-loop 1 (SL1) is highly similar to the consensus minimal structure of the HIV-1 TAR element [97], and third, both Tat and HEXIM1 bind CycT1 in a mutually exclusive manner due to the shared CycT1 binding site [98,99]. However, the Tat activation domain (Figure 3C; AD; aa 1–47) has a 10-fold higher affinity for CycT1 [100]. Tat will thus bind to 7SK and CycT1 by displacing HEXIM1 (Figure 3B) [97]. As soon as TAR forms in the nascent viral transcript emerging from RNAPII, the Tat:P-TEFb complex will bind to TAR, resulting in the release of the inhibitory 7SK snRNP. This activates P-TEFb, allowing it to phosphorylate NELF, DSIF, and Ser2 on the RNAPII CTD, thus flipping the switch between RNAPII pausing and elongation [95].

Interestingly, the human T-lymphotropic virus type 1 transcriptional activator Tax also utilizes P-TEFb for viral transcription and displaces P-TEFb from 7SK snRNP through binding CycT1 [101,102], suggesting that P-TEFb liberation from 7SK snRNP could be a common theme developed by different viruses to support their replication in host cells.

## 8. Recruitment of Transcription Factors by Viral Noncoding RNAs

**Figure 4:**
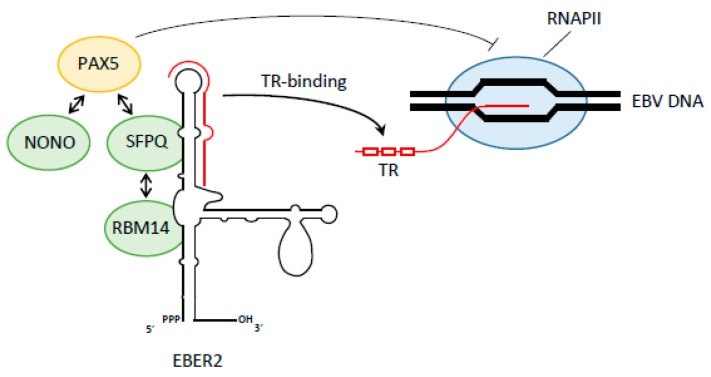
Factors involved in Epstein-Barr virus-encoded RNA (EBV EBER) transcription regulation. RNA structure of EBER2 non coding RNA (black) with the terminal region (TR) binding domain indicated in red. EBER2 recruits several protein factors to the nascent EBV RNA. See the text for more details.

Instead of using promoter-proximal paused transcripts as *cis*-acting scaffolds to recruit TFs, viruses can also use noncoding RNAs. A recent example described that the Epstein-Barr virus (EBV) uses a virally encoded noncoding RNA named EBV-encoded RNA 2 (EBER2) that acts as a *trans*-acting guide to promote its own transcription [75] (Figure 4). EBV is a human γ-herpesvirus that persists as a mini-chromosome in the nucleus of B lymphocytes [103]. Herpesviruses are large enveloped ds DNA viruses that encode more than 80 genes. They are classified into three subfamilies (α-, β-, and γ-herpesviruses). EBV is the causative agent of mononucleosis and is associated with several types of tumors, including lymphomas and carcinomas [104]. Two long ncRNAs called EBER1 (167 nt) and EBER2 (173 nt) are expressed in infected cells at high levels (EBER1: ~10^6^ copies; EBER2: ~2.5 × 10^5^) [105,106] and localize exclusively to the nucleoplasm [107]. Due to their high copy number, these ncRNAs were used as diagnostic tool for EBV infection [108], but their function remained unknown. Employing a technique named ‘capture hybridization analysis of RNA targets’ (CHART), Lee et al. [75] found that EBER2 localizes to the terminal repeats (TRs) in the latent EBV genome. TRs are tandem direct repeats of approximately 550 bp that flank both ends of the linear genome present in virions and are the site of circularization to form the viral episome after its entry into the host cell [109]. EBER2 localizes in the vicinity of a binding site for the paired box protein 5 (PAX5) host transcription factor [110]. This co-localization suggested that these factors collaborate in some way, and Lee et al. show that EBER2 interacts with PAX5, albeit indirectly through an as-yet unidentified bridging factor. PAX5 is known to downregulate viral latent membrane protein 1/2A/2B (LMP1/2) gene expression and entry into EBV lytic replication [111,112]. Top candidates for this bridging factor include three host proteins: splicing factor proline and glutamine rich (SFPQ), non-POU domain-containing octamer-binding protein (NONO), and RNA-binding motif protein 14 (RBM14) [112]. SFPQ and RBM14 contact EBER2 directly as shown by RNA-protein crosslinking, and binding studies show that SFPQ and NONO associate with PAX5 (Figure 4). EBER2 depletion abrogates PAX5 recruitment to the TR, but PAX5 depletion has no effect on EBER2 localization, suggesting that this ncRNA is used to recruit PAX5 to the TR. Indeed, EBER2 base pairs with nascent transcripts generated from the TR regions, thus potentially targeting the interacting PAX5 TF to its consensus sequence sites within the TRs and facilitating its binding.

## 9. Transcription Termination

The last stage of transcription is termination, which leads to the release of the RNA transcript and the dissociation of RNAP from the template DNA. Upon encountering sequence-encoded termination signals (e.g., the canonical polyadenylation signal, AAUAAA), factors will be recruited that cleave and modify the RNA 3′ end. Eukaryotic mRNA undergoes multiple types of modifications at its 3′ end throughout its life cycle [113]. For example, newly synthesized mRNA acquires a long poly (A) tail (up to ∼250 nt) through canonical transcription-coupled polyadenylation, which facilitates mRNA export from the nucleus [114,115]. In the cytoplasm, poly(A) tails are associated with poly(A)-binding proteins (PABPCs) that stabilize the mRNA by acting as a safeguard against multiple decay machineries and promote protein synthesis [116,117]. mRNAs carrying short poly(A) tails (<25 nt) are recognized and poly-uridylated by terminal uridylyl transferases (TUTases) 4 and 7, which labels the mRNA for degradation [118].

Many +ssRNA viruses lack a poly (A) tail at the 3′ end of their genome, but are still efficiently translated. For instance, flaviruses (e.g., dengue virus, West Nile virus) have a capped RNA genome which contains conserved sequences at the 5′ and 3′ ends, allowing for circularization and efficient translation [119,120]. Other examples that follow the same strategy include rotaviruses [121], barley yellow dwarf virus [122], and possibly hepatitis C virus [123].

Additionally, premature transcription termination events are being uncovered that allow viruses to produce another layer of transcripts encoded by the same genome. By using these sub-genomic transcripts, viruses can create regulatory RNA molecules that undergo a different fate than the main vRNA. This can for instance be miRNA production by retroviruses [6] or the above described EBER small ncRNAs produced by EBV. Retroviruses have to produce an alternative transcript as a miRNA-source to prevent cleavage of the vRNA genome during miRNA processing by the Microprocessor complex [6,124]. Most of these sub-genomic transcripts are RNAPIII-encoded [125,126], but can also result from transcription termination of promoter-proximal paused RNAPII [127]. This nascent RNA will subsequently be exported to the cytoplasm, where it can enter the RNAi pathway at the Dicer step.

## 10. Viral Nucleases Induce mRNA Degradation in the Host

Viruses can downregulate host transcription by targeted mRNA degradation, to benefit their own gene expression. In this way, the host transcription machinery is deprived of targets and the virus can redirect the machinery to its own viral expression. As a beneficial side-effect to the virus, lowering host transcription will also reduce the effectiveness of the immune system. Therefore, during infection with many human viruses, the accumulation of viral proteins is accompanied by a progressive global reduction in the production of host proteins, a phenomenon that has been termed “host shutoff”. Viral factors that can induce host shutoff include severe acute respiratory syndrome (SARS) coronavirus nsp1 [128,129], herpes simplex virus 1 (HSV-1) vhs [130,131], vaccinia encoded decapping nucleases D9 and D10 [132,133,134], and two recently described viral endonucleases: Kaposi’s sarcoma-associated herpesvirus (KSHV) SOX [135,136] and Influenza A Virus (IAV) PA-X protein [137].

Like all influenza viruses, IAV is a segmented negative strand RNA virus. The PA-X protein-encoding gene overlaps with the open reading frame (ORF) for the PA FluPol subunit [138]. Ribosomal frameshifting on a rare CGU codon results in a fusion protein with the 191-aa N-terminal mRNA endonuclease domain of PA and an alternative C-terminal X domain of either 41 or 61 aa [139,140]. Consequently, PA-X lacks the CTD of PA responsible for its interaction with the PB1 subunit of FluPol, and was found to share sequence similarity with the above described viral host shutoff proteins that trigger RNA degradation.

KSHV is a γ-herpesvirus that causes tumors of endothelial cells and B cells. During lytic infection with KSHV, global degradation of host RNA is triggered by a virally encoded protein named shutoff and exonuclease (SOX) [135,141,142]. SOX cleaves most cellular mRNAs at random positions [136,143,144]. Although the α-herpesvirus vhs and γ-herpesvirus SOX proteins are not orthologs, they have a similar mechanism of action [144]; both are RNA endonucleases that cut host mRNAs into fragments. The initial cleavage is followed by degradation of the RNA body by the cellular 5′-3′ exonuclease XrnI and potentially other enzymes [136,143]. This mechanism is shared by other viruses with shutoff-RNases like PA-X for IAV and nsp1 for SARS [137,144,145]. Moreover, all known viral host shutoff-RNases, degrade mRNAs transcribed by the cellular RNAPII complex and spare ncRNAs transcribed by RNAPI and RNAPIII [137,143,144].

Notably, viral mRNA transcription escapes from decay-induced repression, and this escape also relies on XrnI. However, the mechanisms that allow viral mRNAs, unlike cellular mRNAs, to escape from XrnI cleavage are unknown.

## 11. Perspective

A complex interplay between viruses and the host transcription machinery that is mediated by nascent RNA has recently become increasingly apparent. Here, we explored a few of the myriad pathways by which viruses (i) use their own nascent RNA—in *cis* or in *trans*—as a scaffold to recruit host transcription factors; (ii) use host-capped RNAs as primers for RNA synthesis; (iii) use termination of nascent RNA synthesis to create sub-genomic mRNAs, and (iv) and down-regulate host nascent RNA production for their own benefit.

Viral RNA synthesis pathways are promising targets for antiviral development because these mechanisms are essential for virus replication. However, in order to identify additional weak spots in viral infection programs, it is important to understand how viruses regulate their gene expression and to disentangle the complex ways in which these pathways are intertwined with host transcription. Many antivirals display adverse side-effects because the viral target molecule and process was incompletely understood. The recently described new insights into the roles of nascent RNA in viral gene regulation are important steps in the path to new, more effective antivirals with fewer side-effects.
